# Analysis of growth in female patients with pediatric-onset systemic lupus erythematosus

**DOI:** 10.1186/1546-0096-9-S1-O19

**Published:** 2011-09-14

**Authors:** NC Mak, C Carbone, LS Lim, DM Levy, ED Silverman, S Kamphuis

**Affiliations:** 1 Sophia’s Children Hospital, Erasmus MC Rotterdam, Netherlands; 2 The Hospital for Sick Children, Toronto, Canada

## Background

Systemic Lupus Erythematosus (SLE) is a lifelong disease characterised by multi-organ involvement and autoantibodies. It is assumed that growth impairment is common in pediatric-onset SLE (pSLE), but studies reporting on the frequency and characteristics of growth abnormalities in pSLE are lacking.

## Aim

To analyse growth in female patients with pSLE and examine its relationship to disease characteristics and treatment.

## Methods

Prospectively collected digital growth curves and menarche status of an inception cohort of 196 female patients with pSLE diagnosed <16 years and a minimal disease duration of 3 years were analysed. Abnormal growth was defined as i) one or more periods of growth arrest (<2 cm) lasting minimal 6 months, ii) more than one growth curve percentile difference between height at diagnosis and at any follow up visit, or iii) < 2 cm growth after menarche. 67 patients were not further analyzed as they had reached final height prior to diagnosis of pSLE.

## Results

58/129 (45%) pSLE patients with growth potential had abnormal growth. The median growth curve percentile at diagnosis (50^th^) was significantly different from the percentile at last follow up (10^th^) in these patients with abnormal growth (p<0.001). Patients with abnormal growth were diagnosed significantly younger than patients with normal growth (9.4±2.8 versus 11.6±2.6 years, p<0.001). 90% of these patients showed period(s) of growth arrest, whereas 10% showed a gradual decline in growth only. The median height of the 36 patients with abnormal growth having reached age 16 years (presumed final height) was significantly lower compared to the height of patients diagnosed after 16 years (154.8±8.6 versus 161±6.1cm, p<0.001). Ethnicity, disease activity over time and cumulative medication use are currently being tested for their potential relation to growth impairment.

## Conclusion

Growth was impaired in approximately half of female pSLE patients and may lead to abnormally low final height. Abnormal growth was characterized by periods of normal growth and growth arrest rather than gradual decline in growth, likely related to disease flares and/or treatment.

